# Genome Wide Transcriptome Analysis of Dendritic Cells Identifies Genes with Altered Expression in Psoriasis

**DOI:** 10.1371/journal.pone.0073435

**Published:** 2013-09-09

**Authors:** Kata Filkor, Zoltán Hegedűs, András Szász, Vilmos Tubak, Lajos Kemény, Éva Kondorosi, István Nagy

**Affiliations:** 1 Institute of Biochemistry, Biological Research Centre of the Hungarian Academy of Sciences, Szeged, Hungary; 2 Institute of Biophysics, Biological Research Centre of the Hungarian Academy of Sciences, Szeged, Hungary; 3 Zenon Bio Ltd., Szeged, Hungary; 4 Creative Laboratory Ltd., Szeged, Hungary; 5 Department of Dermatology and Allergology, University of Szeged, Szeged, Hungary; 6 Dermatological Research Group of the Hungarian Academy of Sciences and the University of Szeged, Szeged, Hungary; INSERM-Université Paris-Sud, France

## Abstract

Activation of dendritic cells by different pathogens induces the secretion of proinflammatory mediators resulting in local inflammation. Importantly, innate immunity must be properly controlled, as its continuous activation leads to the development of chronic inflammatory diseases such as psoriasis. Lipopolysaccharide (LPS) or peptidoglycan (PGN) induced tolerance, a phenomenon of transient unresponsiveness of cells to repeated or prolonged stimulation, proved valuable model for the study of chronic inflammation. Thus, the aim of this study was the identification of the transcriptional diversity of primary human immature dendritic cells (iDCs) upon PGN induced tolerance. Using SAGE-Seq approach, a tag-based transcriptome sequencing method, we investigated gene expression changes of primary human iDCs upon stimulation or restimulation with *Staphylococcus aureus* derived PGN, a widely used TLR2 ligand. Based on the expression pattern of the altered genes, we identified non-tolerizeable and tolerizeable genes. Gene Ontology (GO) and Kyoto Encyclopedia of Genes and Genomes (Kegg) analysis showed marked enrichment of immune-, cell cycle- and apoptosis related genes. In parallel to the marked induction of proinflammatory mediators, negative feedback regulators of innate immunity, such as TNFAIP3, TNFAIP8, Tyro3 and Mer are markedly downregulated in tolerant cells. We also demonstrate, that the expression pattern of TNFAIP3 and TNFAIP8 is altered in both lesional, and non-lesional skin of psoriatic patients. Finally, we show that pretreatment of immature dendritic cells with anti-TNF-α inhibits the expression of IL-6 and CCL1 in tolerant iDCs and partially releases the suppression of TNFAIP8. Our findings suggest that after PGN stimulation/restimulation the host cell utilizes different mechanisms in order to maintain critical balance between inflammation and tolerance. Importantly, the transcriptome sequencing of stimulated/restimulated iDCs identified numerous genes with altered expression to date not associated with role in chronic inflammation, underlying the relevance of our *in vitro* model for further characterization of IFN-primed iDCs.

## Introduction

Plants and animals are constitutively exposed to various pathogens present in the environment including commensal and pathogenic microorganisms. Despite the constant presence of pathogens, infections still develop infrequently, partly because all multicellular organisms have evolved defense mechanisms to combat against harmful microbes [1]. As the inflammation causes dramatic changes in tissue physiology, inflammatory responses must be strictly regulated otherwise uncontrolled inflammation leads to serious pathologic conditions (eg. septic shock, autoimmunity, atherosclerosis) [2]. Numerous regulatory mechanisms, such as the production of anti-inflammatory cytokines or the induction of negative feedback regulators of the TLRs, i.e. members of the SOCS family [3,4] or TNFAIP3 [5] have been described that are preventing the host from the harmful side effects of uncontrolled inflammation.

These molecules also have pivotal role in the long-lasting hyporesponsiveness of the cells and organisms to prolonged/repeated LPS stimulation, a phenomenon called LPS tolerance [6,7]. For a long time LPS tolerance was thought to be a consequence of receptor desensitization [8,9] and it has only recently been demonstrated that transient silencing of pro-inflammatory genes at chromatin level has essential role in the maintenance of LPS tolerance [10]. Foster et al. treated primary mouse macrophages with LPS once or repeatedly and monitored the relative gene expression of inflammatory genes. They found that cytokines such as IL-6 or IL-1β were upregulated after the first LPS stimulation, however they were not re-induced or induced to a much lesser degree after the second LPS challenge, a phenotype defined as tolerizeable. In contrary to cytokines, the relative gene expression of antimicrobial peptides (i.e. cathelicidin-related antimicrobial peptide) proved to be inducible after the second LPS treatment, a phenotype defined as non-tolerizeable. More accurate analysis of these phenotypes demonstrated that covalent histone modifications play important role in the maintenance of LPS tolerance. Even though promoters of both tolerizeable and non-tolerizeable genes were inducibly acetylated, a hallmark of transcriptional activity, in naïve macrophages, only the non-tolerizeable promoters were re-acetylated after LPS treatment in tolerant cells [10]. These data present a novel mechanism for the regulation of LPS tolerance, yet, little is known about transcriptional diversity of PGN tolerance.

In order to get a global view on the gene expression changes of primary human immature dendritic cells (iDCs) upon stimulation or restimulation with *Staphylococcus aureus* derived PGN, a widely used TLR2 ligand, we have performed SAGE-Seq analysis [11]. Based on the expression pattern of the altered genes, we identified non-tolerizeable and tolerizeable genes; a finding resembling that of LPS tolerance [10]. GO gene set enrichment and Kegg pathway analysis showed marked enrichment of immune-, cell cycle- and apoptosis related genes. We also demonstrate, that the expression pattern of TNFAIP3 and TNFAIP8, to date not associated with psoriasis, is altered in both lesional, and non-lesional skin of psoriatic patients. Finally, we show that pretreatment of immature dendritic cells with anti-TNF-α inhibits the expression of IL-6 and CCL1 in tolerant iDCs and partially derepresses TNFAIP8 expression. Importantly, based on the results obtained using the model of PGN induced tolerance and subsequent transcriptome analysis, we were able to identify genes with altered expression in inflammatory diseases such as psoriasis (this study) and TNBS-induced rat model of colitis (unpublished), which underlies the relevance of the *in vitro* model for further characterization of IFN-primed iDCs.

## Results and Discussion

### PGN stimulation and restimulation differentially alters the global gene expression profile of primary human iDCs

**Figure 1:**
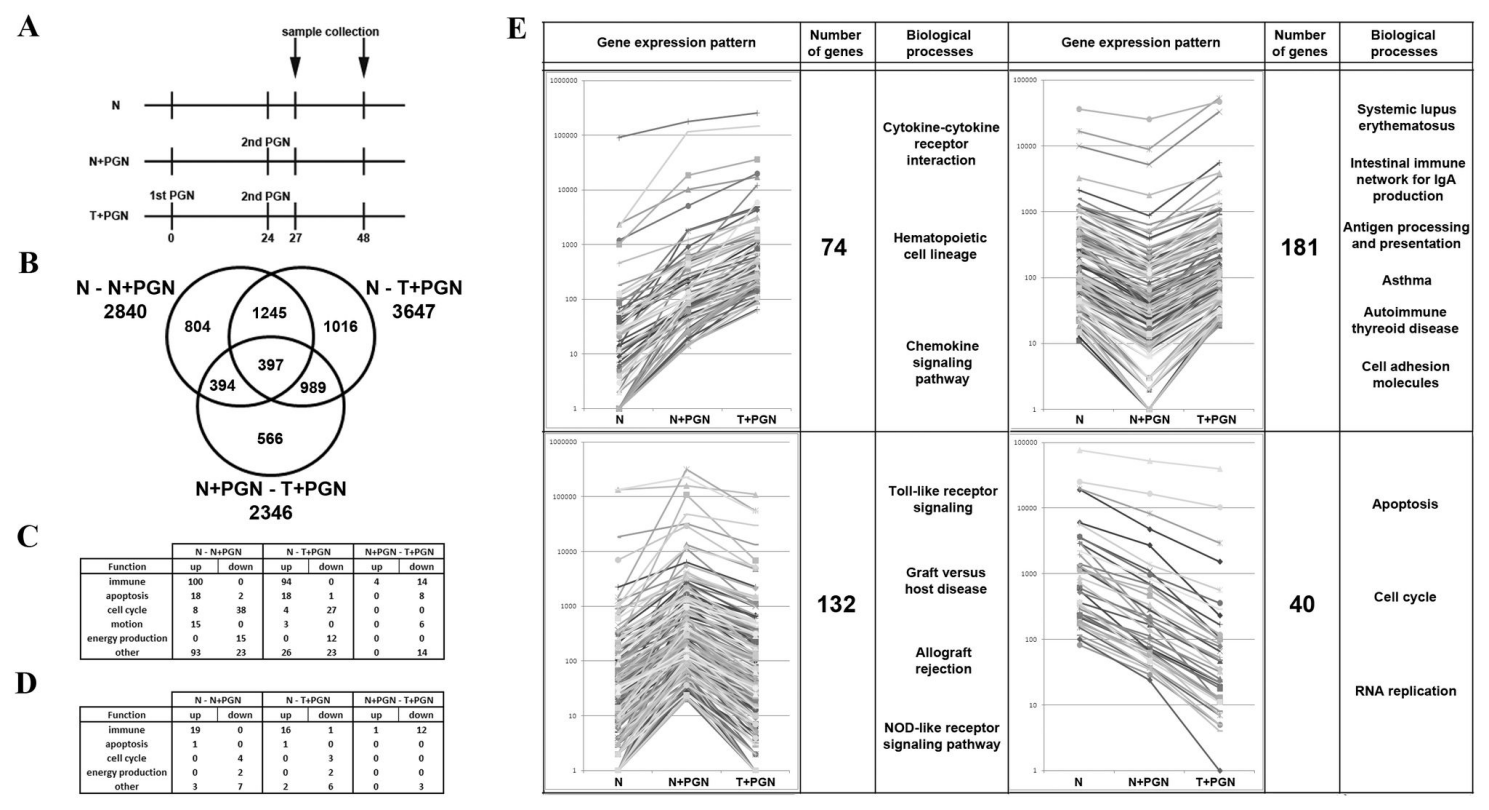
Identification of differentially expressed genes upon PGN stimulation and re-stimulation of immature dendritic cells. (A) Dendritic cell stimulation and sample collection. Immature dendritic cells were left unstimulated (naïve iDCs; N), stimulated with PGN (induced iDCs; N+PGN) or re-stimulated with PGN (tolerant iDCs; T+PGN) mimicking acute or chronic infection, respectively. Total RNA and cell culture supernatants were harvested at 27 and 48 hours post first PGN induction. (B) Venn diagram summarizing the distribution of the 5411 differentially expressed genes (p>0.95, calculated by the Bayesian approach described by *Lash et al* [12].) identified by SAGE-Seq; each cycle represents the pairwise comparison indicated. (C) Gene Ontology and (D) Kegg pathways enriched for genes differentially expressed in the given pairwise comparison. The numbers denotes the cumulative number of genes detected for a particular GO or Kegg term. (E) Based on the classification of genes as non-tolerizeable (genes inducible in tolerant cells) and tolerizeable (genes not inducible in tolerant cells) by Foster et al. [10] four such gene expression patters were identified upon SAGE-Seq analysis comparing naïve vs. induced or tolerant iDCs.

Based on the LPS tolerance model [10] we have first established a model for acute and chronic PGN stimulation, in which we treated primary human immature dendritic cells (iDCs) once or repeatedly (stimulation/restimulation) with *Staphylococcus aureus* derived peptidoglycan (PGN). For the clarity, throughout the manuscript we will use terms induced or N+PGN and tolerant or T+PGN for PGN stimulated or restimulated iDCs, respectively. Next, in order to get a global view on the gene expression pattern of induced or tolerant primary human iDCs (Figure 1A), SAGE-Seq experiments were performed.

In our experiments we are purifying monocytes from buffy-coats of healthy blood donors and are subsequently differentiating them into immature dendritic cells. Since there are a limited number of monocytes in the buffy-coat, we only have a limited number of dendritic cells at the end of differentiation process. Moreover, dendritic cells are non-dividing cells: these features restrict the amount of purified total RNA per sample. Taking all these facts together we opted for pooling RNA samples, isolated from three independent experiments, in equimolar concentration and subsequently sequenced the pooled RNA: this experimental setup ensured the biological heterogeneity of the samples. Even though this experimental design, which is pooling of input RNA, does not permit the estimation of expression variation within treatments, it does facilitate the determination of the fold-change expression changes between treatments. By applying a Bayesian statistical method for testing significance, which is not based on sample variance, but rather on the probability density functions defined by the number of the observed sequenced tags [12], we have altogether identified 5411 genes with significantly altered expression pattern after PGN stimulation/restimulation (Figure 1B). Between naïve (N) and induced (N+PGN) iDCs 2840 genes were found which had significant changes in their expression pattern. Importantly, 804 out of 2840 genes were uniquely characteristic for this category. PGN restimulation altered the expression pattern of 3647 genes (comparison of naïve (N) and tolerant (T+PGN) iDCs)), with 1016 uniquely altered genes. Finally, the gene expression of 566 out of 2346 genes uniquely changed between induced (N+PGN) and tolerant (T+PGN) iDCs. We hypothesized that genes differentially altered in this latter comparison may represent the differences between acute and chronic *Staphylococcal* infection.

To characterize the potential relevance of the alterations resulted by PGN stimulation/restimulation, pathway and gene set enrichment analysis was carried out using Kegg (Kyoto Encyclopedia of Genes and Genomes) database (Figure 1D and Table S2) and the Biological Processes (BiolProc) domain of Gene Ontology Project (Figure 1C and Table S1). Although Kegg allows for a more accurate analysis by providing consistent and standardized annotations by linking individual genes to components of the Kegg biochemical pathways [13] we used both pathway analysis tools to reduce the possibility of false results. Both PGN stimulation and restimulation induced massive enrichment of both BiolProc GO terms and Kegg pathways, particularly from the functional groups immune response, apoptosis and cell cycle (Figure 1C, D and Table S1, S2). These results are not surprising, taking into account the fact that iDCs are known to rapidly respond to immune challenge [14], in this case PGN. However, when comparing the gene expression patterns of induced vs. tolerant iDCs, both pathway analysis tools highlighted significant downregulation of a number of, primarily, genes from the immune response functional group (Figure 1C, D and Table S1, S2). These results suggest the induction of tolerogenic mechanism after persistent PGN stimulation similarly to that observed in a model of LPS tolerance [10].

Based on the results of the pathway analysis and taking into account the specific feature of the SAGE-Seq technology, namely that NlaIII - the enzyme used to fragment mRNA - has recognition site of approximately every 200-300 nucleotides in the human genome, we investigated the expression pattern of individual sequence tags. This latter represents the short subsequence of an mRNA and is used to identify gene transcripts in SAGE-Seq experiments. Foster et al. have recently used an LPS induced model of tolerance in murine macrophages and have shown that TLR-induced genes fall into non-tolerizeable and tolerizeable categories [10]. We were particularly interested if these categories can be identified in our model of PGN induced tolerance and have thus specifically screened sequence tag patterns for the four categories shown on Figure 1E. Indeed, 427 sequence tags fall in one out of four monitored categories (Figure 1E), a finding resembling that of LPS tolerance [10]. In the first two categories, PGN stimulation leads to the upregulation of 255 out of 427 tested tags, in contrast, two other categories are comprised of those 172 tags that are downregulated in induced iDCs. Expression pattern of the tested tags (206 up- and 221 downregulated tags) in tolerant iDCs further subdivides these categories. Here we describe the four monitored categories in more detail. The first category is comprised of those 74 tags out of 427 tested where the upregulation in induced iDCs as compared to naïve cells is observed, and these tags are even further upregulated in tolerant iDCs (Figure 1E, upper panel). Notably, genes of the immune response GO term and Kegg pathways belonging to eg. cytokine and chemokine signaling biological processes, fell into this category. In contrast, 181 tags were significantly downregulated in tolerant iDCs, as compared to their induction in induced iDCs: these tags belong to the second category where an enrichment of genes from the Toll-like receptor signaling pathway is observed (Figure 1E, second panel). These data are in complete agreement with those observed in a model of LPS tolerance, where these two categories are designated as class non-tolerizeable and tolerizeable, respectively [10]. 132 tags are classified into the third category, which is comprised of genes showing down-regulation in induced and upregulation in tolerant iDCs, such as those of the antigen processing and presentation pathway (Figure 1E, third panel). Finally, the remaining 40 tags show further downregulation in tolerant iDCs, as compared to that observed in induced cells, eg. apoptosis and cell cycle related genes (Figure 1E, bottom panel).

Together with results collected using the model of LPS tolerance, our data on PGN tolerance further strengthens the view that even attenuated signaling through pattern recognition receptors, such as TLRs, is sufficient for the induction of a number of genes [10]. In the course of our study, we were primarily interested to identify those genes that show significantly altered gene expression pattern when comparing induced and tolerant iDCs, as we hypothesize that this approach may facilitate the identification of genes responsible for the development and maintenance of chronic inflammation. SAGE-Seq experiments indeed indicate that our *in vitro* experimental model provides valuable tool for identifying differences at the gene expression level between acute and chronic infection.

### Stimulation and restimulation of iDCs with PGN differentially alters the expression of immune related genes

Since SAGE-Seq approach does not allow the determination of exact relative gene expression values, just the direction of altered expression (i.e. up- or downregulation) we decided to more accurately examine the expression levels of selected molecules. For this, and based on SAGE-Seq and subsequent pathway analysis and enrichment studies we have chosen members of the immune response, apoptosis, and cell cycle functional groups in order to more accurately analyze their relative gene expression and secreted protein level by QRT-PCR and ELISA, respectively.

**Figure 2:**
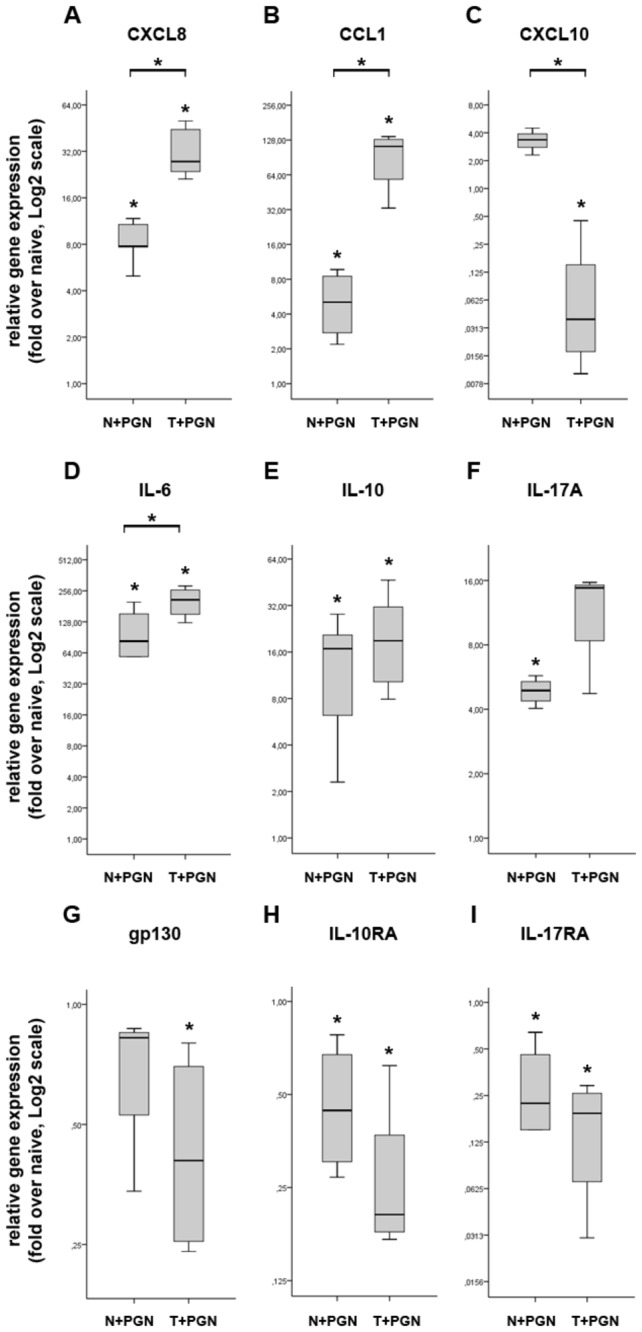
Different expression pattern of pro-inflammatory mediators and receptors in induced and tolerant iDCs at 27h. The relative gene expression of pro-inflammatory effectors such as CXCL8 (A), CCL1 (B), CXCL10 (C), IL-6 (D), IL-10 (E) and IL-17A (F) shows significant upregulation in induced (N+PGN) as compared to the naïve (N) iDCs. The expression of CXCL8 (A), CCL1 (B), IL-6 (D) and IL-17A (F) shows further upregulation in tolerant iDCs (T+PGN) as compared not only to the naive but to the induced iDCs as well. In contrast, the expression of CXCL10 (C) in tolerant iDCs is significantly downregulated when compared both to naïve and induced iDCs. In contrast to the effector molecules, receptors involved in inflammation such as gp130 (G), IL-10RA (H) and IL-17RA (I), were significantly down regulated in both induced and tolerant iDCs as compared to naïve cells. The ratio of each mRNA relative to the 18S rRNA was calculated using the 2^-ΔΔCT^ method. Data are representative of 3 or more independent experiments and are presented as interquartile range (box) with median (horizontal black bar) and minimum and maximum values. The significance of differences between sets of data was determined by Student’s paired t-test using SPSS Statistics; *p<0.05.

PGN stimulation resulted in significant relative gene expression upregulation of the majority of investigated immune effector molecules such as CXCL8, CCL1, CXCL10, IL-6, IL-10 and IL-17A (Figure 2A–F) compared with naïve cells. Furthermore, all except CXCL-10 showed significant further relative gene expression upregulation in tolerant iDCs; these five are classified as non-tolerizeable genes. Surprisingly, although IL-10 has primarily anti-inflammatory role, we detected its significantly increased expression in both induced and tolerant iDCs (Figure 2E), suggesting that both pro- and anti-inflammatory mechanisms are simultaneously induced upon PGN stimuli. Importantly, the expression of IFN-γ inducible CXCL10 in tolerant iDCs was completely abolished, thus it belongs to class tolerizeable genes (Figure 2C).

We also aimed to determine the impact of PGN stimulation/restimulation on the relative gene expression pattern of selected cytokine receptors. Our data demonstrate that the expression of CCR8 (receptor for CCL1; data not shown), gp130 (receptor for IL-6; Figure 2G), IL-10RA (receptor for IL-10; Figure 2H) and that of IL-17RA (receptor for IL-17A; Figure 2I) all show significant dowregulation in both induced and tolerant iDCs. This opposite expression pattern of cytokines and their respective receptors suggest a paracrine role for the induced cytokines, which is in agreement with the primary function of iDCs playing role in antigen uptake and the recruitment of other effector cells to the site of the infection [15].

**Figure 3:**
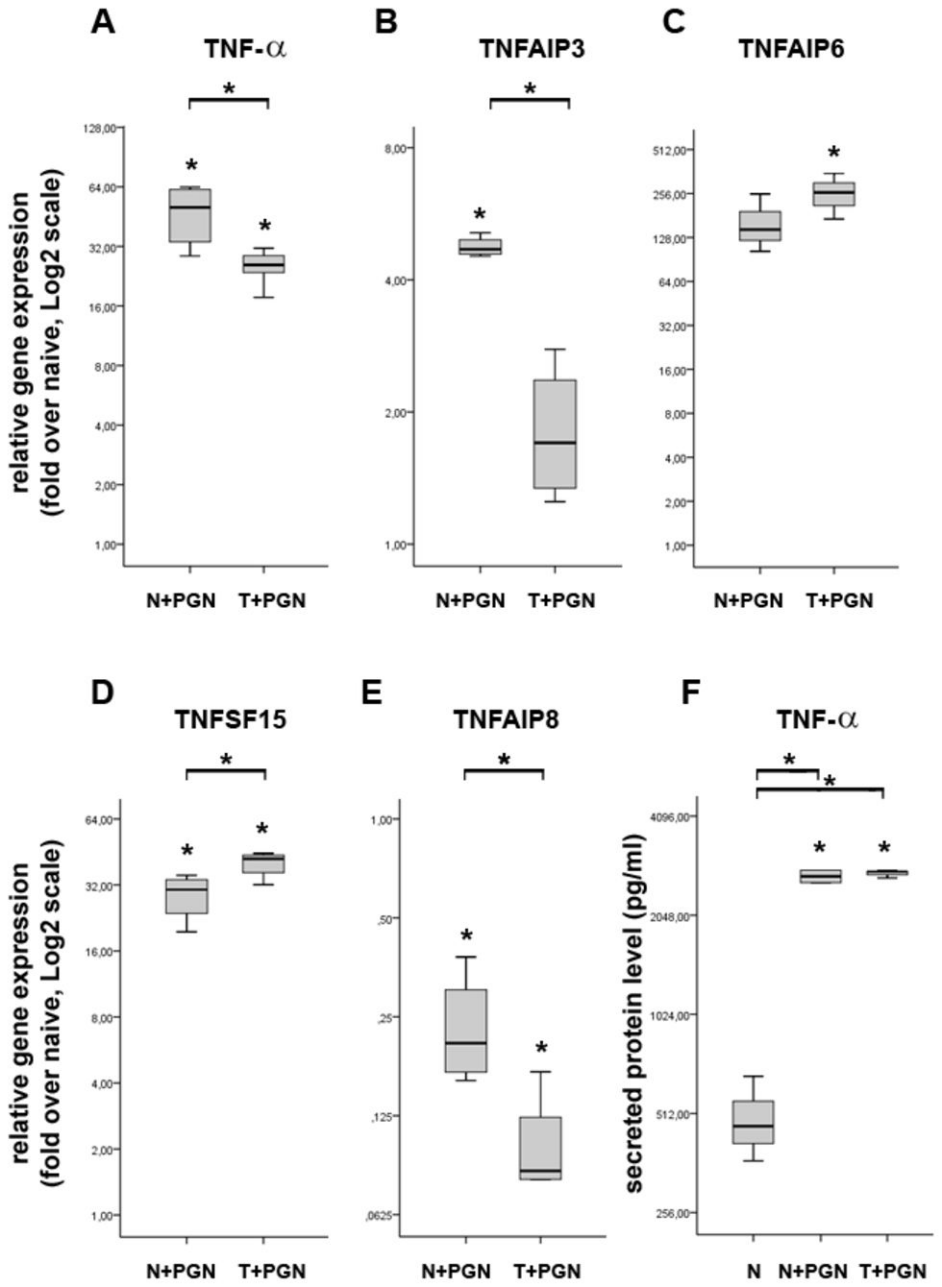
TNF-α and TNF-α regulated genes show distinct expression pattern in induced and tolerant iDCs at 27h. The relative gene expression of pro-inflammatory effectors such as TNF-α (A), TNFAIP3 (B), TNFAIP6 (C) and TNFSF15 (D) shows significant upregulation in induced iDCs as compared to naive cells. The expression of TNFAIP6 and TNFSF15 shows further upregulation in tolerant iDCs as compared to the induced cells (C, D), in contrast, the expression of TNF-α (A) and TNFAIP3 (B) in tolerant iDCs are significantly downregulated when compared to induced iDCs. TNFAIP8 (E) is significantly downregulated in both induced and tolerant iDCs. (F) Secreted TNF-α protein level was determined from cell culture supernatants by ELISA at 48h. In accordance with relative gene expression results, both stimulation and restimulation of iDCs with PGN induces significant TNF-α protein secretion. The ratio of each mRNA relative to the 18S rRNA was calculated using the 2^-ΔΔCT^ method. Data are representative of 3 or more independent experiments and are presented as interquartile range (box) with median (horizontal black bar) and minimum and maximum values. The significance of differences between sets of data was determined by Student’s paired t-test using SPSS Statistics; *p<0.05.

**Figure 4:**
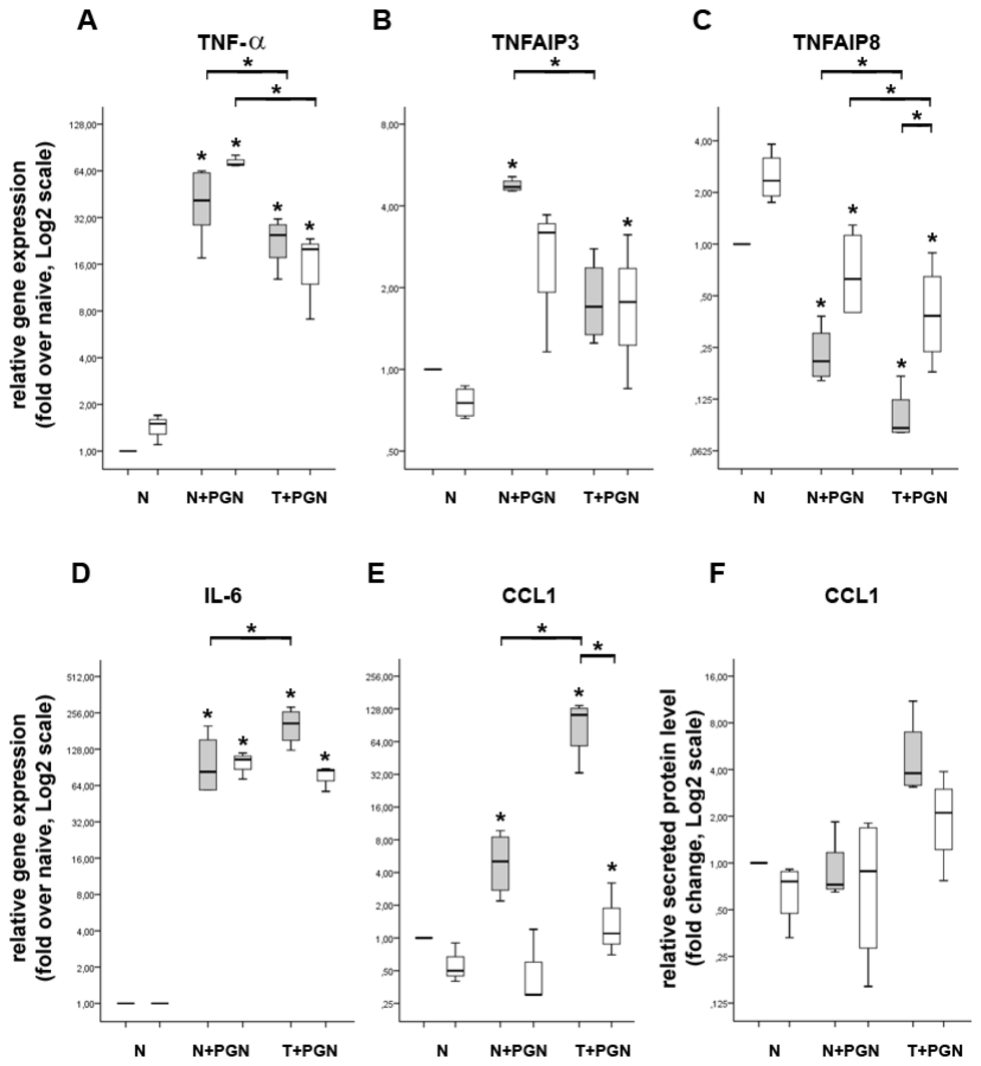
Targeting TNF-α with infliximab decreases the expression of IL-6 and CCL1 in tolerant iDCs at 27h. Soluble chimeric monoclonal anti-TNF-α antibody (infliximab) was added to the culture supernatant in a therapeutic concentration (white bars) and the effects on gene and protein expression were monitored by QRT-PCR (A–E) and ELISA (F). Samples without infliximab treatment (gray bars) were used as controls. While infliximab pre-treatment has no effect on the expression of TNF-α (A), TNFAIP3 (B), TNFAIP8 (C), IL-6 (D) and CCL1 (E, F) in naïve and induced iDCs, it partially releases the inhibition of TNFAIP8 (C) and significantly dowregulates the expression of IL-6 (D) and CCL-1 (E, F) in tolerant iDCs. The ratio of each mRNA relative to the 18S rRNA was calculated using the 2^-ΔΔCT^ method. Data are representative of 3 or more independent experiments and are presented as interquartile range (box) with median (horizontal black bar) and minimum and maximum values. The significance of differences between sets of data was determined by Student’s paired t-test using SPSS Statistics; *p<0.05.

SAGE-Seq experiments identified numerous members of the TNF-α related genes being differentially expressed between induced and tolerant iDCs (data not shown). Since TNF-α is, together with IL-1α and IL-6, an acute-phase molecule having pivotal role in the early immune response against invading pathogens, we decided to determine the exact expression pattern of selected members of the family. Our data demonstrates that the robust expression of TNF-α in induced iDCs is abolished in tolerant cells (Figure 3A, F) supporting earlier findings showing its rapid inducibility and fading [16]. The expression pattern of TNFAIP3 follows that of TNF-α, being abolished in tolerant cells, thus both belonging to the class tolerizeable genes. Since anti-TNF-α pretreatment had no significant effect on the expression of these two molecules (Figure 4A, B), we suggest their expression pattern is indeed a direct consequence of PGN stimulation/restimulation and not a secondary effect of *de novo* TNF-α secretion.

Opposed to TNF-α and TNFAIP3, the relative gene expression of TNFAIP6 (TNF-α-stimulated gene 6; also known as TSG6) as well as TNFSF15 (also known as TL1A) was significantly induced in tolerant iDCs (Figure 3C, D). Since both molecules are directly inducible by TNF-α stimulation we sought to determine if their elevated expression in tolerant iDCs is a consequence of TNF-α secretion evoked by PGN induction. Surprisingly, by using anti-TNF-α pretreatment, we were unable to abolish the elevated expression of TNFAIP6 and TNFSF15 in tolerant iDCs (data not shown). As both molecules play important roles in the pathogenesis of autoimmune diseases [17], these results offer an interesting aspect for the partial unsuccessfulness of anti-TNF-α therapies: we suggest that TNF-α is not the only factor triggering the expression of genes classified as TNF-α inducible.

We have also determined that the expression of the negative regulator of innate immunity TNFAIP8 (also known as TIPE2) is significantly downregulated in induced iDCs and further downregulated in tolerant cells (Figure 3E). Recent reports demonstrated that TNFAIP8 is predominantly expressed in immune cells where it negatively regulates T cell receptor (TCR) and TLR signaling [18–20]. The absence of TNFAIP8 expression results in hyper-responsiveness to TCR and TLR activation and enhanced pro-inflammatory cytokine secretion. This is in line with our findings showing that PGN restimulation results in significant gene expression downregulation of TNFAIP8 and, in parallel, upregulation of numerous cytokines and chemokines eg. CXCL8, CCL1, IL-6, IL-17A, TNF-α etc. Finally, our results, showing the release of the TNFAIP8 inhibition and, in contrast, inhibition of IL-6 and CCL1 expression in tolerant iDCs by anti-TNF-α pretreatment (Figure 4C–F) suggests that, apart of PGN, *de novo* synthesized TNF-α indeed regulates the expression of TNFAIP8, IL-6 and CCL1.

**Figure 5:**
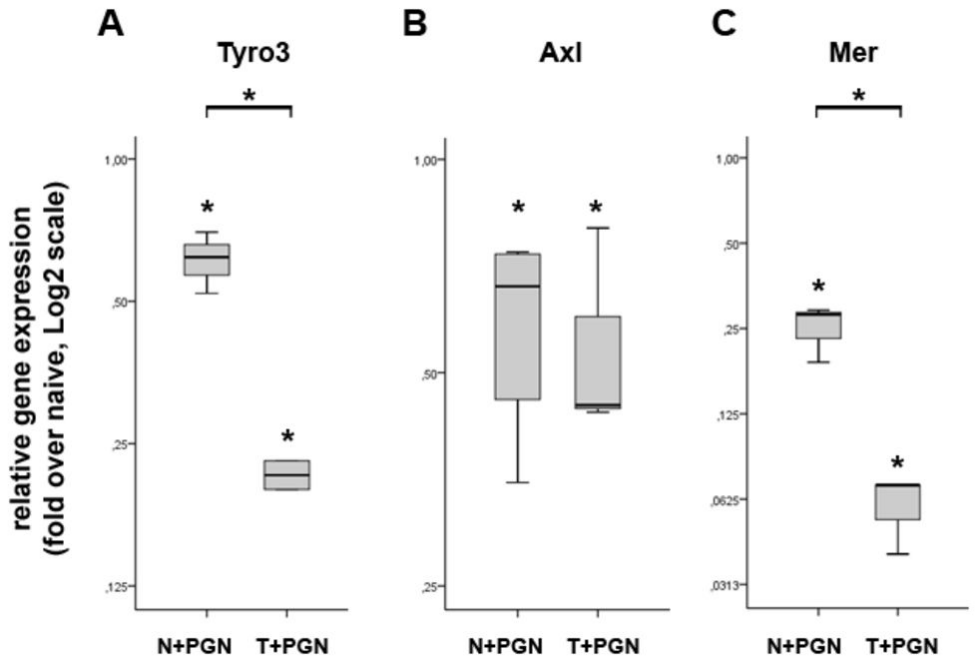
The expression of TAM receptor family in induced and tolerant iDCs at 27h. The relative gene expression levels of Tyro3 (A), Axl (B) and Mer (C) were determined by QRT-PCR. All three genes show significantly dowregulated expression in induced iDCs when compared to naive cells (N+PGN samples). In addition, the expression of Tyro3 and Mer is further downregulated in tolerant iDCs as compared to induced iDCs. The ratio of each mRNA relative to the 18S rRNA was calculated using the 2^-ΔΔCT^ method. Data are representative of 3 or more independent experiments and are presented as interquartile range (box) with median (horizontal black bar) and minimum and maximum values. The significance of differences between sets of data was determined by Student’s paired t-test using SPSS Statistics; *p<0.05.

Similarly to TNFAIP8, TAM family of receptor tyrosine kinases, consisting of Tyro3, Axl and Mer, play central role in the intrinsic inhibition of the inflammatory response to pathogens [21]. Rothlin et al. have reported that TAM activation of murine DCs inhibits the secretion of effector molecules, such as cytokines and chemokines [22]. Notably, in our experimental model, the expression of all three molecules is significantly downregulated in both induced and torelant iDCs (Figure 5A–C); in addition, the expression of Tyro3 and Mer is further downregulated in tolerant iDCs (Figure 5A and C). This is in line with the observed elevated level of cytokine and chemokine gene expression; moreover these data are in accordance with the recently reported marked downregulation of TAM receptor tyrosine kinases in psoriatic epidermis [23].

In order to determine if the identified gene expression patterns are specific to the given sampling time point, that is at 27h post first PGN stimulation, we have analyzed the expression pattern of seven genes at 48h post first PGN stimulation (Figure S1, S2). The results show, that all but TNFAIP3 retained their expression pattern detected at 27h post first PGN stimulation, although with a slightly altered kinetics.

### PGN stimulation downregulates the expression of cell cycle- and apoptosis-related genes

**Figure 6:**
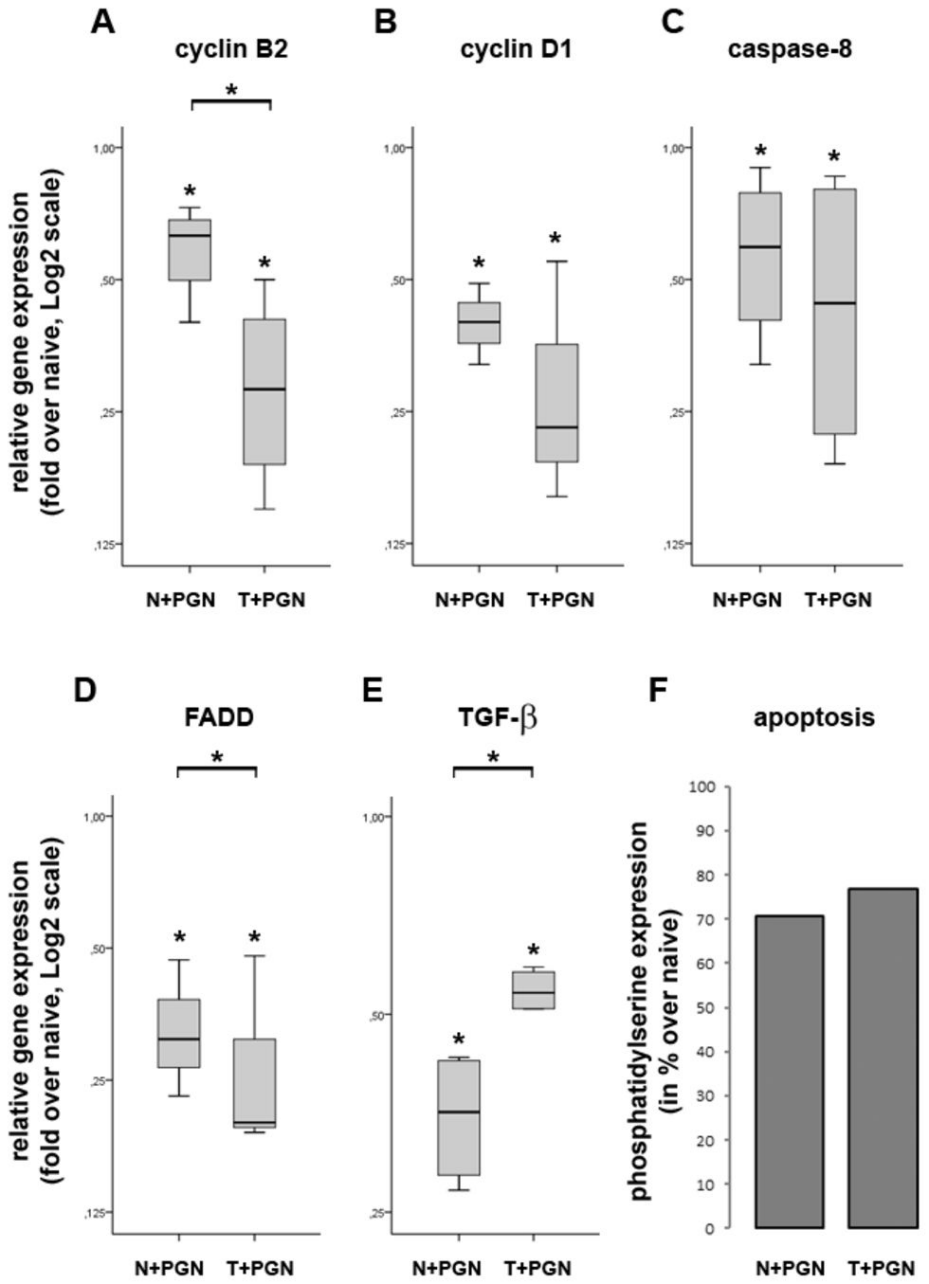
The expression of cell cycle and apoptosis regulators in induced and tolerant iDCs at 27h. Based on the SAGE-Seq data, we have chosen 2 cell cycle regulators, cyclin B2 (A) and cyclin D1 (B), and three apoptosis regulators, caspase-8 (C), FADD (D) and TGF-β (E) for the validation by QRT-PCR. All of the selected genes show significantly dowregulated expression in both induced and tolerant iDCs when compared to naive cells. In addition, while still downregulated relative to naive cells, the expression of TGF-β in tolerant iDCs is significantly upregulated as compared to induced iDCs (E). (F) Induced and tolerant iDCs exhibit decreased apoptosis as determined by fluorescence intensity measurements of phosphatidylserine expressed on cell membrane, measured with Annexin V-Alexa Fluor 488 binding. The ratio of each mRNA relative to the 18S rRNA was calculated using the 2^-ΔΔCT^ method. Data are representative of 3 or more independent experiments and are presented as interquartile range (box) with median (horizontal black bar) and minimum and maximum values except for the phosphatidylserine expression intensity (F). The significance of differences between sets of data was determined by Student’s paired t-test using SPSS Statistics; *p<0.05.

The strict regulation of cell division cycle has fundamental role in the maintenance of tissue homeostasis. As cyclins and cyclin-dependent kinases regulate the transition of different phases of cell cycle and, based on pathway analysis of SAGE-Seq data, numerous members seem to be suppressed in both induced and tolerant iDCs (Figure 1E) we examined the impact of PGN stimulation on the gene expression of cyclin B2 (CycB2; Figure 6A) which is involved in G2-M transition and cyclin D1 (CycD1; Figure 6B) which has central role in G1-S transition [24]. QRT-PCR approach indeed corroborated SAGE-Seq data, demonstrating that PGN stimulation/restimulation resulted in significant relative gene expression downregulation of both CycB2 and CycD1. These findings, together with the fact that DCs are non-dividing cells, suggest that cross-linking of immune receptor with its respective ligand, in this case TLR2-PGN, induces maturation process which is characterized by functional differentiation of the antigen presenting cells without numeral expansion.

The induction of apoptotic processes has important role in the maintenance of the transient nature of the immune response [25,26]. Pathway analysis, showing the enrichment of apoptosis related genes in our model (Figure 1C–E), prompted us to investigate the impact of PGN stimulation on the relative gene expression of three molecules involved in the regulation of cell fate and apoptosis. We have determined, that caspase-8, Fas-associated protein with death domain (FADD) and TGF-β are all significantly downregulated upon PGN stimulation (Figure 6C–E). While we could not detect any difference in the suppression of caspase-8 and FADD gene expression between induced and tolerant iDCs, the suppression of TGF-β in tolerant iDCs is significantly lower. Since the downregulation of proapoptotic genes does not necessarily imply resistance to apoptosis, we sought to define the apoptosis of induced and tolerant iDCs. We monitored the amount of phosphatidylserine expressed on cell membrane in the early phase of apoptosis, and found reduced expression in both induced and tolerant iDCs as compared to naïve cells (Figure 6F). The role of programmed cell death is not limited to the maintenance of tissue homeostasis, it has pivotal role in preventing the organism against the development of autoimmune diseases [27]. Hence, by dowregulating this pathway tolerant iDCs may play a role in the development of autoimmune diseases. In addition, our data show strong correlations with earlier findings showing that the down regulation of FADD enhances the relative gene expression of IL-1β, IL-6, IL-10 and TNF-α in mouse epidermis [5].

These data suggest that acute and persistent *Staphylococcal* infection inhibits cell division cycle by downregulating the expression of cyclins, moreover, the reduced expression level of proapoptotic genes suggests the induction of prosurvival mechanisms which may, in turn, lead to prolonged antigen presentation and the elimination of invading pathogens.

### The expression pattern of TNF-α related genes from psoriatic skin shows similar expression pattern with that observed in our in vitro model

We have determined, that pro- and anti-inflammatory molecules maintain critical balance during acute and chronic PGN stimuli to successfully eliminate invading pathogens and to prevent the host against the harmful side effects of uncontrolled inflammation. Although the pathogenesis of psoriasis has not been fully understood yet, the immunological basis of this disease has been well demonstrated [28]. It is well known that the enhanced level of proinflammatory mediator TNF-α has pivotal role in the pathogenesis of psoriasis [29]. In addition, a previous report demonstrated strong nuclear immunopositivity for TNFSF15 in psoriatic skin samples, in contrast, TNFSF15 was absent in healthy skin samples [17]. However, little is known about the expression pattern of other TNF-α related genes in the course of this autoimmune disease. In order to determine the relative gene expression pattern of TNFAIP3, TNFAIP6 and TNFAIP8 in plaque-type psoriasis we have performed QRT-PCR experiments on psoriatic non-lesional and lesional dermal and epidermal samples. As controls, skin biopsy samples form healthy individuals were used.

**Figure 7:**
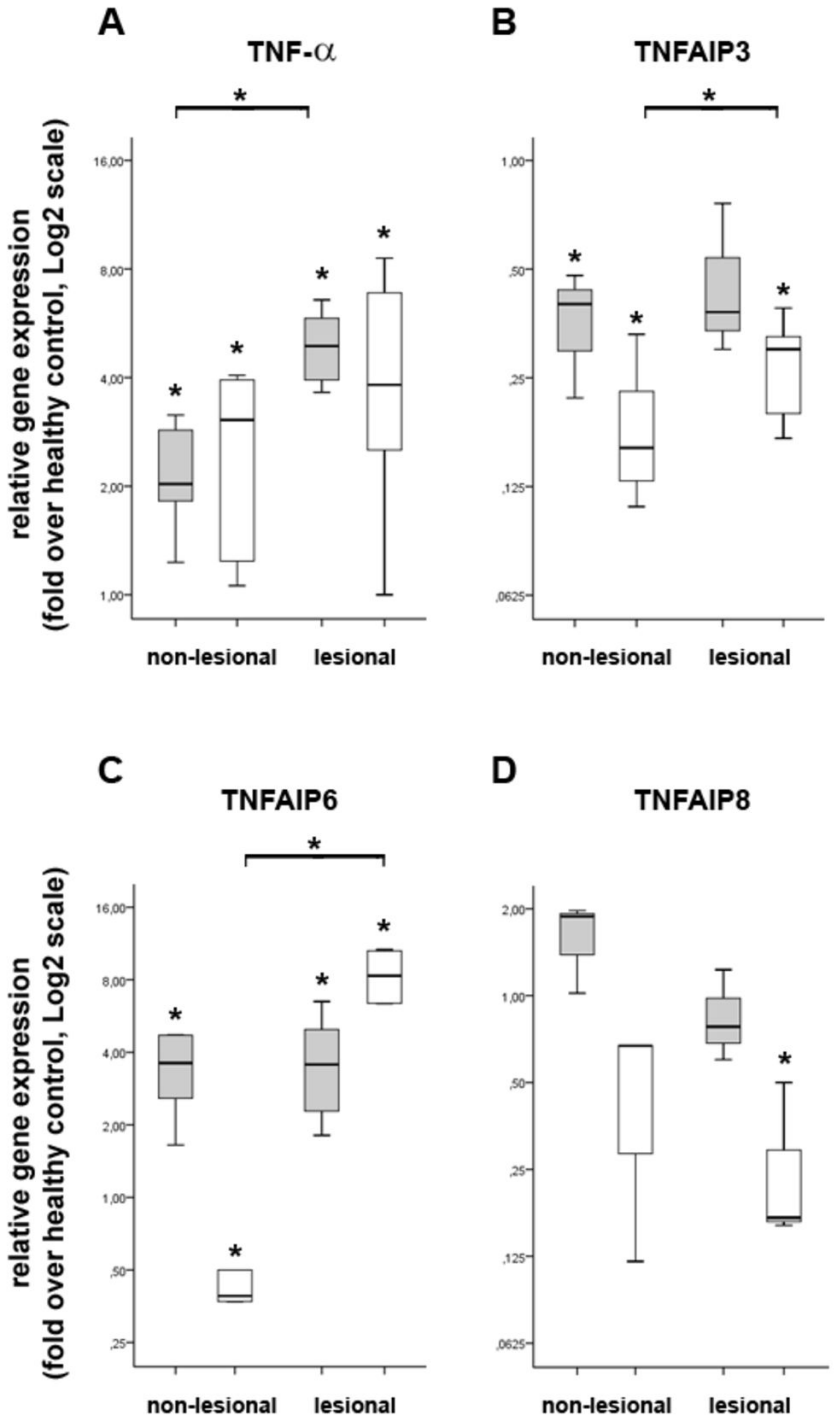
The expression level of selected genes in psoriasis. Shave biopsy specimens were taken from healthy individuals (controls without psoriasis; n=3) and from psoriatic patients (n=3): both lesional and non-lesional samples were taken from this latter group. RNAs were isolated from epidermis (grey box) and dermis (white box), the gene expression levels were determined by QRT-PCR and represented relative to healthy skin. We have detected significant induction in the expression of TNF-α (A) in both non-lesional and lesional skin of psoriatic patients. Similar expression pattern was observed in case of TNFAIP6 (C), although its expression in non-lesional epidermis shows significant downregulation. In contrast, TNFAIP3 (B) and TNFAIP8 (D) show significant downregulation. Data are representative of 3 or more individuals; *p<0.05.

First, we examined the relative gene expression pattern of TNF-α. In agreement with earlier studies we found that the relative gene expression level of TNF-α is upregulated in psoriatic non-lesional epidermal and dermal samples compared with healthy samples (Figure 7A). Furthermore, in lesional samples a more robust upregulation was detected as compared to non-lesional counterparts. As these data, together with those showing elevated expression of CXCL-8 in lesional epidermis [30], corroborate earlier findings [31] we used these samples for further experiments.

In accordance with PGN restimulated iDCs (Figure 3C) the relative gene expression of TNFAIP6 is significantly upregulated in psoriatic non-lesional and lesional epidermal samples compared to healthy skin (Figure 7C; gray bars). In non-lesional dermal samples significant gene expression downregulation was detected as compared to healthy skin, in contrast, we detected robust gene expression upregulation in lesional samples (Figure 7C; open bars). In contrary to the pro-inflammatory mediators, the anti-inflammatory effector molecules, TNFAIP3 and TNFAIP8 (Figure 7B and D, respectively) were both downregulated in psoriatic non-lesional and lesional epidermal and dermal samples as compared to healthy controls. Again, these results show strong correlations with those obtained in PGN restimulated iDCs (Figure 3B and E, respectively). Of note, TNFAIP3 deficient mice die early after the birth due to serious multi-organ inflammation [32] and the targeting of TNFAIP3 in different cell types was shown to be associated with chronic inflammation and autoimmunity [33–35]. Furthermore the expression of TNFAIP3 in peripheral blood mononuclear cells of psoriatic patients negatively correlates with disease severity [36], and TNFAIP3 gene has been identified as a susceptibility locus for several autoimmune disorders, including psoriasis [37]. TNFAIP8 is also an essential negative regulator of inflammation [18] and is also involved in the protection against apoptosis [38]. To the best of our knowledge this is the first report demonstrating the significant downregulation of TNFAIP3 and TNFAIP8 in psoriatic dermis and epidermis which may explain the enhanced cytokine production and the decreased sensitivity to pro-apoptotic processes characteristic for psoriasis.

Collectively, we have successfully used the results obtained on tolerant iDCs to identify genes with altered expression pattern in psoriatic skin. Our results suggest that beside the enhanced level of TNF-α, the elevated expression of TNFAIP6 also has important role in the maintenance of chronic inflammation which is characteristic for psoriasis. Furthermore, the downregulation of negative regulators TNFAIP3 and TNFAIP8 (this study), as well as members of the TAM receptor family [30] in psoriasis, suggest that negative feedback regulators of inflammation are disturbed in psoriasis which may, partly, explain chronic inflammation.

### Targeting TNF-α with infliximab derepresses TNFAIP8 and decreases the expression of IL-6 and CCL1 in tolerant iDCs

Based on the more specific understanding of the pathogenesis of skin inflammatory conditions at a molecular level, the inhibition of pro-inflammatory mediators with antibodies or soluble receptors became the mainstream therapy to treat dermatologic diseases, such as psoriasis or atopic dermatitis [39,40]. As TNF-α plays central role in the pathogenesis of psoriasis it represents an active target for biological therapies, hence, numerous TNF-α blocking agents are used to treat psoriasis. Even though TNF-α is thought to be an instigator of inflammation, little is known about downstream molecular effect of TNF-α blocking. Our results, demonstrating that the expression pattern of tolerant iDCs can be used for identification of genes with altered expression in chronic inflammatory conditions such as psoriasis (see above) or colitis (unpublished) prompted us to test the impact of TNF-α deprivation at cellular level. The application of infliximab did not altered TNF-α and TNFAIP3 gene expression (Figure 4A, B) suggesting that their expression in our model is TNF-α independent. Infliximab did, however, markedly reduced the relative gene expression of IL-6 in tolerant iDCs (Figure 4D). This is in good agreement with earlier findings showing that infliximab significantly reduced the relative gene expression of acute-phase proteins such as IL-1β and IL-6 [41]. Moreover, we observed significant derepression of TNFAIP8 gene expression in tolerant cells (Figure 4C) suggesting that, at least partially, TNF-α directly represses its expression.

CCL1 inflammatory chemokine plays a central role during the recruitment of activated T cells and CCR8^+^ DCs to the site of skin inflammation [42]. In order to determine if the significantly induced CCL1 expression in tolerant DCs (Figure 2B) is TNF-α dependent we again used infliximab pretreatment. Indeed, infliximab abolished CCL1 gene expression and protein secretion (Figure 4E, F, respectively) in tolerant iDCs indicating that TNF-α plays a major role in PGN-induced CCL1 expression. This is in agreement with the finding showing that TNF-α induces the expression of CCL1 in macrophages [43]. Taking into account recent reports demonstrating elevated CCL1 expression is atopic dermatitis [42,44] our results may provide an additional explanation for the successful application of anti-TNF-α therapy in atopic dermatitis [40]: by targeting TNF-α the downstream expression of CCL1 is decreased attenuating thereby the initiation and amplification of atopic skin inflammation. 

## Conclusions

This study utilized transcriptomic data obtained from the model of PGN tolerance in primary human immature dendritic cells (iDCs) in order to obtain a list of putative candidate genes differentially expressed upon acute and chronic infection. Based on their expression pattern, 427 sequence tags clustered in one of four monitored categories with remarkable expression differences observed when comparing induced vs. tolerant cells. Our findings suggest that after PGN stimulation/restimulation the host cell utilizes different mechanisms in order to maintain critical balance between inflammation and tolerance leading to the elimination of impending pathogens and protecting the host against harmful side effects resulting from uncontrolled inflammation. Importantly, a selection of genes with differential expression in induced and/or tolerant iDCs shows similar expression pattern in chronic inflammatory conditions, such as psoriasis (this study and [30]) or TNBS-induced colitis in rats (unpublished) underlying the relevance of the *in vitro* model for further characterization of IFN-primed iDCs.

## Materials and Methods

### Monocyte isolation and dendritic cell differentiation

Buffy coats of healthy blood donors were obtained from the Hungarian National Blood Transfusion Service, Szeged, Hungary. Importantly, to limit the person-to-person variability as much possible, we used buffy-coats of Rh+ type A group blood donors (A+) in all our experiments. From these preparations peripheral blood mononuclear cells (PBMCs) were separated by Ficoll Paque Plus (GE Healthcare) density gradient centrifugation as described previously [45]. In brief, monocytes were isolated by adherence on tissue culture plastic plates. Immature dendritic cells (iDCs) were obtained by culturing monocytes for five days in RPMI-1640 medium (Gibco) supplemented with 10% heat-inactivated FBS (Gibco), 1% penicillin/streptomycin solution (Gibco), 1000 units/ml recombinant human granulocyte-macrophage colony stimulating factor (GM-CSF, Sigma) and 1000 unit/ml recombinant human interferon-α (IFN-α, Sigma). Cells were cultured at 37^o^C in an atmosphere of 5% (v/v) CO_2_ in air.

### iDC stimulation and sample collection

iDCs were treated with 10μg/ml *Staphylococcus aureus* (*S. aureus*) derived peptidoglycan (PGN, Sigma) according to the model described in Figure 1. Briefly, iDCs were left untreated (naïve, N), stimulated (induced; N+PGN) or restimulated (tolerant; T+PGN) with 10µg/ml PGN, in order to mimic acute or chronic infection. In some experiments, soluble chimeric monoclonal anti-TNF-α antibody (infliximab, Schering-Plough) was added to primary monocytes in parallel with GM-CSF and IFN-α. Infliximab has a long half-life of approximately 10 days, which ensured the neutralization of all secreted TNF-α protein during iDC generation. Moreover, freshly prepared infliximab was added to the culture supernatants along with PGN stimulations in order to neutralize *de novo* synthesized and secreted TNF-α. Total RNA and cell culture supernatants were collected at 27 and 48 hours post first PGN treatment, that is 3 and 24 hours post second PGN treatment.

### Quantitative reverse transcriptase polymerase chain reaction (QRT-PCR)

Total RNA was extracted from iDCs using RNeasy Plus Mini Kits (Qiagen) according to the manufacturer’s instructions. The quality and quantity of extracted RNA was determined using NanoDrop (Thermo Scientific), Qubit (Life Technologies) and Bioanalyzer (Agilent) measurements. cDNA was synthesized from at least 100ng of total RNA by using the High Capacity RNA to cDNA Kit (Life Technologies) according to the manufacturer’s instructions. SybrGreen and TaqMan technology based real-time quantitative PCR was used to quantify the relative abundance of the selected mRNAs. For this, specific exon spanning gene expression assays were used; TaqMan assay IDs and primer sets are listed in Tables 1 and 2, respectively. As controls, we used reaction mixtures without cDNA. All of the measurements were performed in duplicate with at least three biological replicates. The ratio of each mRNA relative to the 18S rRNA was calculated using the 2^-ΔΔCT^ method.

**Table 1:** TaqMan assays used in QRT-PCR experiments.

**Gene**	**Assay number**
18S rRNA	Hs99999901
IL-6	Hs00174131_m1
IL-10	Hs00961522_m1
CXCL8	Hs00174103_m1
TNF-α	Hs00174128_m1
TNFAIP6	Hs01113602_m1
TNFAIP8	Hs00226190_m1
Tyro3	Hs00170723_m1
Axl	Hs00242357_m1
Mer	Hs01031979_m1

**Table 2:** SybrGreen primer sets used in QRT-PCR experiments.

**Gene**	**Forward (5' -3')**	**Reverse (5' -3')**	**product length (bp)**
CXCL10	TTGTTCCACGTGTTGAGATCATTG	GCAGCCCTCTGTGTGGTCCATC	188
CCL1	CTTCACCAGGCTCATCAAAGCTG	TCTGGAGAAGGGTACCTGCAT	150
gp130	TCCCTGCCTGTGACTTTCAAGGG	AGGTCCTTTGGAAGGTGGAGCTTG	140
IL-10RA	GTCAGTCCCAGCCCAAGGGTAG	AGCTCTGTCCCATGAGCGTCTG	154
IL-17A	ATCACAATCCCACGAAATCCAG	CTTTGCCTCCCCAGATCACAGAG	207
IL-17RA	GCATCACCACAGGCGGTGG	AGGGATGGGGCTTGGGCAGGT	100
TNFAIP3	GCTGAAAACGAACGGTGACGG	AGAGACTCCAGTTGCCAGCGG	159
TGF-β	TGTCACCGGAGTTGTGCGG	GGCCGGTAGTGAACCCGTTGATG	131
CycB2	TGGCTCCAAAGGGTCCTTCTCCC	CTGCAGAGCTGAGGGTTCTCCCA	139
CycD1	TCAAGTGTGACCCGGACTGCCT	ACGTCGGTGGGTGTGCAAGC	160
CASP8	AACAGCTTCAGAAGAAGGAGCAG	AGGTTCAAGTGACCAACTCAAGGGG	101
FADD	CTGGGGAAGAAGACCTGTGTGCAG	GCTCTGTCAGGTTGCGGGGG	144

### RNA extraction from skin biopsy samples

Shave biopsies from non-lesional and lesional skin of psoriatic patients as well as healthy control skin biopsies were collected as described previously [46]. After removal of the subcutaneous tissue, skin biopsies were incubated overnight at 4°C in Dispase solution (Roche Diagnostics). On the following day, the epidermis was separated from the dermis and samples mounted in 1ml of TRIzol Reagent (Life Technologies) to which 400µl chloroform (Sigma) was added and vortexed vigorously. Samples were centrifuged at 13000rpm for 10 minutes and the upper phase was loaded onto gDNA eliminator spin columns of the RNeasy Plus Mini Kits (Qiagen). Subsequent RNA extraction and quantification as well as cDNA synthesis and QRT-PCR was performed as described above.

All tissue samples were taken with the patient’s written informed consent and the approval of the University of Szeged Ethical Committee. The study was conducted according to the Declaration of Helsinki Principles.

### SAGE-Seq

Buffy-coats of healthy individuals were obtained at three different occasions, and subsequent monocyte-isolation, dendritic cell differentiation and finally the RNA isolation was performed in three different experiments, as described above. SAGE-Seq [11] was performed using SOLiD SAGE kit (Life Technologies) according to the manufacturer’s instructions. Briefly, purified total RNA from three independent experiments, representing three healthy A+ blood-donors, was pooled in equimolar ratios and was bind to Dynabeads Oligo(dT) EcoP magnetic beads in order to capture polyadenilated RNAs. cDNA was synthesized on the magnetic beads by using SuperScriptIII Reverse Transcriptase and *E. coli* DNA polymerase. Next, NlaIII restriction was performed in order to generate GTAC containing sticky end at the complementary DNA strand. Barcode adaptor A was ligated, which contains an EcoP15I restriction enzyme recognition site and a truncated internal adaptor sequence. An EcoP15I digestion was carried out to cleave the construct off from the magnetic beads. EcoP15I restriction also resulted in 2 base-pair long overhanging tag. Adaptor B was joined to the 5’ end of each tag which contains the P1 primer binding site. Each library was amplified in an emulsion PCR, sequencing beads deposited on sequencing slide and sequenced on SOLiD V4 System (Life Technologies).

It is important to emphasize, that due to the limited amount to input RNA, we pooled RNA from three independent experiments, subsequently, SAGE sequencing was performed on one RNA pool per each of the three experimental groups.

### Data availability

Short-read archive of the three sequenced libraries was deposited in NCBI’s Short Read Archive at http://www.nvbi.nlm.nih.gov/sra/ under accession SRA076165.

### Bioinformatic analysis of SAGE-Seq

For mapping of SAGE-Seq tags to the human transcriptome we created virtual libraries containing all the possible 18 bases length sequences of ENSEMBL human transcript data set (version: GRCh37.58) located next to an NlaIII restriction site. Virtual tag libraries and the pre-processed and quality filtered SAGE-Seq libraries were uploaded into an in house developed data warehouse [47]. Mappings were performed by the relational database engine of the data warehouse in a way that only perfect matches over the entire 22 bp length of the 18 bp tag plus the 4 bp NlaIII recognition site were allowed. During the mapping process the system tracked the so-called multiple mapping events where tags detected in the experiments could be assigned to more than one transcript. Statistical comparison of SAGE-Seq data from different samples was performed using the Bayesian method described by Lash et al. [12]. Briefly, a key-by-key comparison of two key-count distributions was performed by generating a probability that the frequency of any key in the distribution differs by more than a given fold factor from the other distribution. The algorithm performs the statistical analysis by taking into account the potential difference in the total size of the tag libraries compared. In our analysis we used a 2-fold factor difference of transcript expression level as the subject of the Bayesian statistical evaluation. According to the suggestion of Chen et al. [48] the c parameter value of simplified beta distribution function, that corresponds to parameters a and b of the original function, was set to 2. The algorithm returns a probability value (p) for each tag describing the chance that the detected count numbers represent a fold difference of the tag concentration between the investigated samples greater than or equal to 2. The change of a tag expression was accepted as significant if *p* was above 0.95.

### Gene enrichment studies

For uncovering the biological processes represented in the massive output of the high-throughput SAGE-Seq experiments we executed gene enrichment studies using gene ontology (GO) [49] and Kegg [50] pathway databases as the source of gene annotation information. The extraction of the overrepresented annotation categories, and the underlying statistical calculations were performed by DAVID, a high-throughput data-mining software tool. For the enrichment calculations we used the DAVID default population background containing all human genes with at least one annotation in the analyzed categories. To check the significance of gene enrichment, DAVID applies a modified Fisher’s exact test [51]. The enrichment *p*-values produced by the Fisher’s exact test were corrected by Benjamini multiple testing method to control family-wide false discovery rate. Gene annotation categories were accepted to be significantly overrepresented when the associated *p* value was less than 0.05 after Benjamini correction.

### Measurements for secreted cytokine levels

Harvested cell culture supernatants were centrifuged and the concentrations of secreted TNF-α and CCL1 were measured by Quantikine Human Immunoassay Kits (R&D Systems) following the manufacturer’s instructions. Serial dilutions of the respective recombinant human proteins were used to generate standard curves. The optical density of each well was determined using a microplate reader (FLUOstar Optima, BMG Labtech) set to 450nm with a wavelength correction set to 540nm.

### Detection of apoptosis using fluorescent microscopy

Immature dendritic cells were grown on glass coverslips and after appropriate treatments cells were fixed with 4% (w/v) paraformaldehyde for 4 min at room temperature and washed with phosphate-buffered saline (PBS). Cells were incubated with PBS containing 1% (v/v) FBS (Gibco) for 1 hour at room temperature. After washing with Annexin binding buffer (ABB; 10 mM HEPES pH 7.4, 140 mM NaCl, 2.5 mM CaCl_2_), samples were incubated with ABB in the presence of 1:40 diluted Alexa 488-conjugated Annexin V (Invitrogen; Life Technologies) for 30 min and washed twice with ABB. Samples were mounted in Citifluor mounting media (Citifluor) to prevent quenching. The images were captured with an Olympus Fluoview FV1000 confocal laser scanning microscope (Olympus Life Science Europa) and quantified with ImageJ. The extent of apoptosis was calculated from fluorescence intensity as follows: fluorescence intensity of induced or tolerant iDCs / fluorescence intensity of naïve cells and is shown in %.

### Data representation and statistical analysis

Data show median (horizontal black bar), interquartile range (box) and minimum and maximum values. The significance of differences between sets of data was determined by Student’s paired t-test using SPSS Statistics for Windows. A *p* value of less than 0.05 was considered significant.

## Supporting Information

Figure S1 **The expression pattern of proinflammatory mediators and receptors in induced and tolerant iDCs at 48h.** The relative gene expression of pro-inflammatory mediators CXCL8 (A), CCL1(B) and IL-6 (C) remained significantly upregulated at 48h post first PGN treatment. The ratio of each mRNA relative to the 18S rRNA was calculated using the 2^-ΔΔCT^ method. Data are representative of 3 or more independent experiments and are presented as interquartile range (box) with median (horizontal black bar) and minimum and maximum values. The significance of differences between sets of data was determined by Student’s paired t-test using SPSS Statistics; *p<0.05.
(TIF)

Figure S2 **The expression pattern of TNF-α and TNF-α regulated genes in induced and tolerant iDCs at 48h.** The relative gene expression of TNF-α (A) and TNFAIP6 (C) remains upregulated, the expression of TNFAIP8 (D) remains significantly downregulated and there is no change in the expression level of TNFAIP3 (B) at 48h post first PGN treatment. The ratio of each mRNA relative to the 18S rRNA was calculated using the 2^-ΔΔCT^ method. Data are representative of 3 or more independent experiments and are presented as interquartile range (box) with median (horizontal black bar) and minimum and maximum values. The significance of differences between sets of data was determined by Student’s paired t-test using SPSS Statistics; *p<0.05.
(TIF)

Table S1 Significantly enriched biological process gene ontology annotations among transcripts that showed changes in expression upon PGN stimulation and restimulation of iDCs.
(XLS)

Table S2 **Significantly enriched Kegg pathways among transcripts that showed changes in expression upon PGN stimulation and restimulation of iDCs.**
(XLS)
