# Supplementary figures and images for: Genome Wide Transcriptome Analysis of Dendritic Cells Identifies Genes with Altered Expression in Psoriasis
Source: PLoS One. 2013 Sep 9;8(9):e73435. doi: 10.1371/journal.pone.0073435 (PMC3767820; doi:10.1371/journal.pone.0073435)

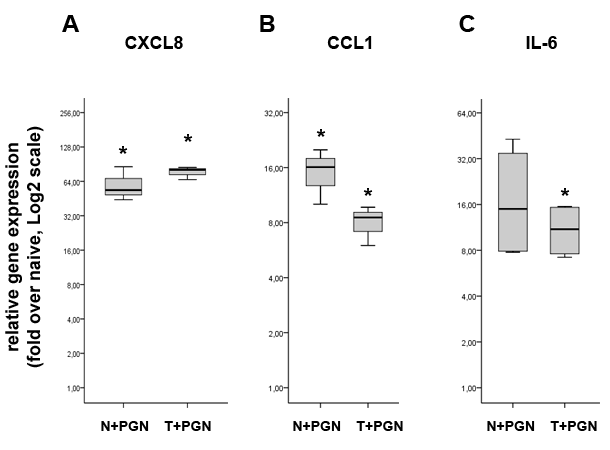

Supplement: Figure S1 — The expression pattern of proinflammatory mediators and receptors in induced and tolerant iDCs at 48h. The relative gene expression of pro-inflammatory mediators CXCL8 (A), CCL1(B) and IL-6 (C) remained significantly upregulated at 48h post first PGN treatment. The ratio of each mRNA relative to the 18S rRNA was calculated using the 2-ΔΔCT method. Data are representative of 3 or more independent experiments and are presented as interquartile range (box) with median (horizontal black bar) and minimum and maximum values. The significance of differences between sets of data was determined by Student’s paired t-test using SPSS Statistics; *p<0.05. (TIF) [file pone.0073435.s001.tif]

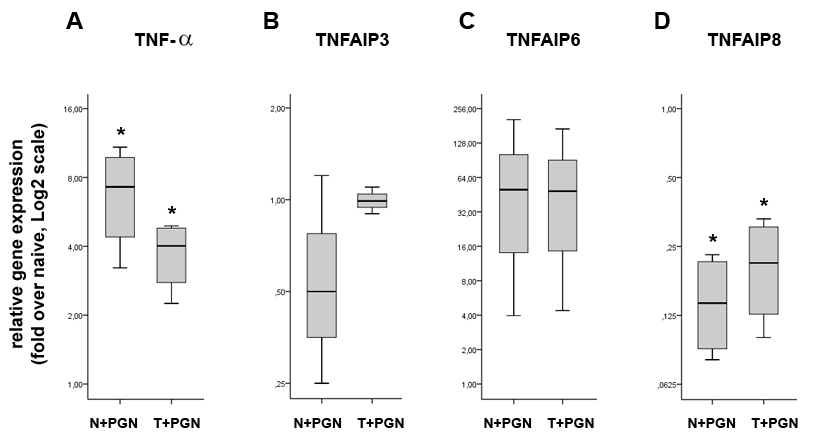

Supplement: Figure S2 — The expression pattern of TNF-α and TNF-α regulated genes in induced and tolerant iDCs at 48h. The relative gene expression of TNF-α (A) and TNFAIP6 (C) remains upregulated, the expression of TNFAIP8 (D) remains significantly downregulated and there is no change in the expression level of TNFAIP3 (B) at 48h post first PGN treatment. The ratio of each mRNA relative to the 18S rRNA was calculated using the 2-ΔΔCT method. Data are representative of 3 or more independent experiments and are presented as interquartile range (box) with median (horizontal black bar) and minimum and maximum values. The significance of differences between sets of data was determined by Student’s paired t-test using SPSS Statistics; *p<0.05. (TIF) [file pone.0073435.s002.tif]
